# Long Noncoding RNA SOX2-OT: Regulations, Functions, and Roles on Mental Illnesses, Cancers, and Diabetic Complications

**DOI:** 10.1155/2020/2901589

**Published:** 2020-11-26

**Authors:** Pu-Yu Li, Ping Wang, She-Gan Gao, Dao-Yin Dong

**Affiliations:** ^1^Department of General Medicine, The First Affiliated Hospital of Henan University of Science and Technology, Luoyang 471000, China; ^2^Department of Public Health, School of Basic Medical Sciences, Henan University of Science and Technology, Luoyang 471000, China; ^3^Henan Key Laboratory of Cancer Epigenetics, The First Affiliated Hospital of Henan University of Science and Technology, Luoyang 471000, China

## Abstract

SRY-box transcription factor 2 (SOX2) overlapping transcript (SOX2-OT) is an evolutionarily conserved long noncoding RNA. Its intronic region contains the SOX2 gene, the major regulator of the pluripotency of embryonic stem cells. The human SOX2-OT gene comprises multiple exons and has multiple transcription start sites and generates hundreds of transcripts. Transcription factors (IRF4, AR, and SOX3), transcriptional inhibitors (NSPc1, MTA3, and YY1), and miRNAs (miR-211 and miR-375) have been demonstrated to control certain SOX2-OT transcript level at the transcriptional or posttranscriptional levels. Accumulated evidence indicates its crucial roles in the regulation of the SOX2 gene, miRNAs, and transcriptional process. Restricted expression of SOX2-OT transcripts in the brain results in the association between SOX2-OT single nucleotide polymorphisms and mental illnesses such as schizophrenia and anorexia nervosa. SOX2-OT is notably elevated in tumor tissues, and a high level of SOX2-OT is well correlated with poor clinical outcomes in cancer patients, leading to the establishment of its role as an oncogene and a prognostic or diagnostic biomarker for cancers. The emerging evidence supports that SOX2-OT mediates diabetic complications. In summary, SOX2-OT has diversified functions and could be a therapeutic target for various diseases.

## 1. Introductions

SRY-box transcription factor 2 (SOX2) overlapping transcript (official symbol SOX2-OT according to the HUGO Gene Nomenclature Committee) is an evolutionarily conserved long noncoding RNA (lncRNA). The SOX2-OT gene is mapped to human chromosomal locus 3q26.33 and is located in a highly conserved region of more than 750 kb in humans and other vertebrates [1]. The SOX2-OT gene contains the key regulator of embryonic stem cell pluripotency, i.e., the SOX2 gene, within its intronic region, and both SOX2-OT and SOX2 are transcribed in the same orientation [2]. The human SOX2-OT gene comprises multiple exons and has multiple transcription start sites with complicated transcriptional features [1, 2]. Initially, Amaral et al. identified several variants of the SOX2-OT gene in mice and humans, including transcripts with multiple transcription start sites [2]. This group also identified SOX2-OT variants in chickens, frogs, and zebrafishes, and some transcripts appear to be species-specific [2]. As deep DNA sequencing technology has advanced, researchers have found that the SOX2-OT gene is expressed as 104 mRNA-like transcripts, the longest of which is approximately 4.3 kb in humans (according to the Ensembl genome database project) [3]. The comprehensive noncoding RNA sequence database RNA Central, which is maintained by the European Molecular Biology Laboratory-European Bioinformatics Institute (EMBL-EBI), includes information for 161 transcripts of the human SOX2-OT gene [4]. In mice, the SOX2 overlapping transcript (official symbol Sox2ot according to Mouse Genome Informatics) is mapped to chromosome 3qA3, and the transcript length is shorter than that of the human homolog. The Ensembl genome database includes 18 information for transcripts of the mouse Sox2ot gene [5].

We searched studies regarding the SOX2-OT gene on PubMed and found that the SOX2-OT gene has received unprecedented attention within the last five years. The PubMed records indicated that only 17 articles exploring the functions of the SOX2-OT gene were published before 2015, whereas nearly 80 articles investigating the SOX2-OT gene were published from 2015 to date (May 2020). The earliest study regarding the SOX2-OT gene was published in 2003 [6]. In this study, the SOX2 gene was discovered by genomic analysis to be located in an intron of another gene, which they named SOX2-OT [6]. The researchers demonstrated that SOX2-OT contains at least five exons (current studies have shown that it contains dozens of exons) and produces a mRNA-like transcript from the same strand that SOX2 is located on [6]. This transcript is evolutionarily conserved; the human SOX2-OT transcript and available mouse expressed sequence tags share 80% nucleotide identity [6]. In addition, the genomic region (approximately 40 kb) encompassing the SOX2-OT transcription unit is highly conserved across vertebrates [6]. Subsequently, accumulating evidence has indicated that the SOX2-OT gene is associated with mental illnesses, cancers, and diabetic complications. SOX2-OT expression is upregulated during the central nervous system development and is restricted to the brain in adult humans and other vertebrates [2, 7]. Therefore, single-nucleotide polymorphisms (SNPs) in the SOX2-OT gene are associated with mental illnesses [8, 9]. Moreover, an increased expression of SOX2-OT is observed in tissues from various cancers; SOX2-OT typically functions as an oncogene to influence cancer progression and can serve as a prognostic or diagnostic biomarker for cancers [10]. In addition, studies have demonstrated that the SOX2-OT gene is involved in diabetic complications and other diseases [11–13]. In this review, we comprehensively summarize the most recent research progress in the regulation and function of SOX2-OT and the association of this lncRNA with various diseases. Moreover, we discuss the potential opportunities and challenges revealed by these findings.

## 2. SOX2-OT Regulates SOX2 Expression

**Figure 1:**
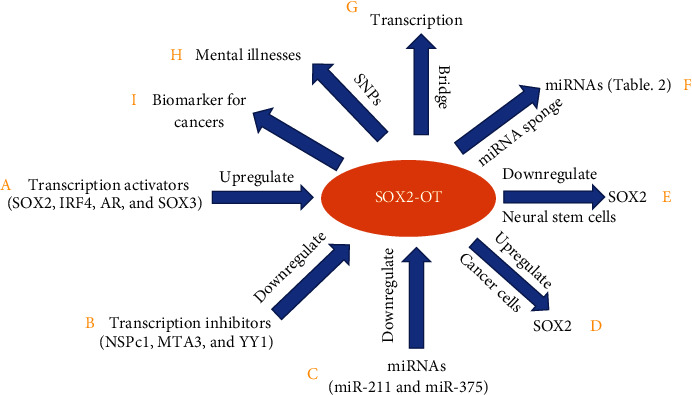
Long noncoding RNA SOX2-OT's regulations, functions, and roles on mental illnesses, cancers, and diabetic complications. Transcription activators ((A) SOX2, IRF4, AR, and SOX3) can upregulate SOX2-OT expression, but transcription inhibitors ((B) NSPc1, MTA3, and YY1) and miRNAs ((C) miR-211 and miR-375) downregulate SOX2-OT expression. D. SOX2-OT upregulates SOX2 expression in cancer cells. (E) SOX2-OT downregulates SOX2 expression in neural stem cells. SOX2-OT can control miRNA levels via serving as a miRNA sponge (F) and affect transcription via serving as a bridge between epigenetic factors and DNA (G). (H) SOX2-OT SNPs are associated with mental illnesses. (I) SOX2-OT is a biomarker for cancers.

The SOX2 gene is a key regulator of stem cell pluripotency and is embedded in an intron of SOX2-OT [1, 2]. lncRNAs can regulate the expression of adjacent overlapping genes via specific mechanisms [14]. Various studies have investigated the regulatory relationship between SOX2-OT and SOX2 (Table 1). Almost all cancer studies involving SOX2-OT and SOX2 have indicated that upregulation of SOX2-OT promotes SOX2 expression in cancer cells (Table 1); however, one study showed that SOX2-OT overexpression did not affect SOX2 expression [15]. Studies on septic cardiomyopathy demonstrated that the level of SOX2-OT is inversely correlated with that of SOX2 [16]. Furthermore, the levels of SOX2-OT and SOX2 are negatively correlated during neural differentiation of mouse embryonic stem cells [7] (Figure 1).

Mechanistic investigations have revealed that SOX2-OT upregulates or downregulates the SOX2 expression through diverse pathways. Two studies demonstrated that SOX2-OT upregulates the SOX2 expression via the miR-200 family members in cancer cells [17, 18]. SOX2-OT acts as a miRNA sponge that competitively binds to miR-200 family members in order to upregulate the expression of SOX2 in cancer cells [17, 18]. One study revealed that the luciferase activity of the SOX2 promoter is significantly increased when SOX2-OT is overexpressed in pancreatic ductal adenocarcinoma cells, suggesting that SOX2-OT is a transcriptional activator of the SOX2 gene [19]. However, a study on central nervous system development showed that SOX2-OT physically interacts with the multifunctional transcriptional regulator YY1, which binds to several CpG islands in the SOX2 locus in a SOX2-OT-dependent manner and downregulates SOX2 expression in neural stem cells [20]. Another study showed that SOX2-OT impairs the formation of the chromatin promoter-enhancer loop upstream of the SOX2 gene and disrupts SOX2 transcription in neural stem cells [7]. Although few studies have investigated the mechanism by which SOX2-OT regulates the SOX2 expression in cancer cells or neural stem cells (Table 1), the regulation of the SOX2 expression by SOX2-OT in tumor cells follows a pattern opposite to that in neural stem cells (Table 1).

## 3. SOX2-OT Is a miRNA Sponge and a Regulator of Transcription

**Figure 2:**
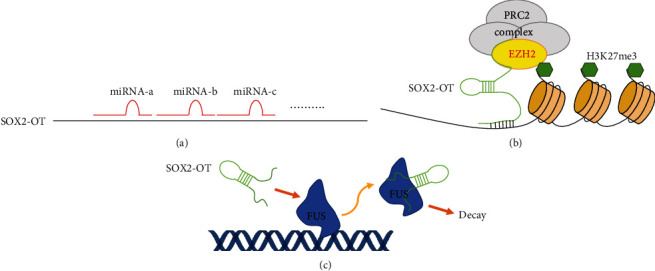
SOX2-OT is a miRNA sponge and a regulator of transcription. (a) SOX2-OT is a miRNA sponge. (b) SOX2-OT acts as a bridge between epigenetic factors and DNA to affect gene expression. (c) SOX2-OT acts as a destabilizer of transcription factors to control gene expression. PRC: polycomb repressive complex; EZH2: enhancer of zeste 2; FUS: FUS RNA binding protein.

Research has suggested that some lncRNAs are involved in the competitive binding of miRNAs [21]. The members of this major subset of lncRNAs are called competing endogenous RNAs (ceRNAs), or miRNA sponges, and they form a regulatory network that controls the expression of protein-coding genes [22]. In this network, lncRNAs positively regulate the expression of protein-coding genes by competitively binding to their miRNAs [22]. SOX2-OT has been identified as an important ceRNA that affects cancer progression (Table 2, Figure 2(a)). An omics study revealed that SOX2-OT interacted with 6 differentially expressed miRNAs (hsa-mir-192-5p, hsa-mir-215-5p, hsa-mir-204-5p, hsa-mir-205-5p, hsa-mir-338-3p, hsa-mir-375) among 96 esophageal squamous cell carcinoma samples and 13 normal tissue samples [23]. In addition, numerous studies have demonstrated that SOX2-OT can bind to unique miRNAs in various cancers, and almost no overlapping miRNAs have been identified among those cancers (Table 2). miR-200c is the only exception, as SOX2-OT can target miR-200c in both bladder cancer and pancreatic ductal adenocarcinoma [17, 18]. Although SOX2-OT can target various miRNAs, it regulates similar cellular functions and behaviors, such as cancer cell proliferation, migration, invasion, metastasis, epithelial-mesenchymal transition (EMT), and stemness maintenance (Table 2).

In addition to acting as a miRNA sponge, SOX2-OT acts as a regulator of transcription by serving as a bridge between epigenetic factors and DNA to affect gene expression (Figure 2(b)). A recent study revealed that SOX2-OT interacts with EZH2, recruits EZH2 to DNA to form the polycomb repressive complex 2 (PRC2), induces H3K27me3, and epigenetically inhibits PTEN expression in laryngeal squamous cell carcinoma cells [24]. Studies have demonstrated that SOX2-OT binds to nervous system polycomb 1 (NSPc1), a key component of polycomb repressive complex 1 (PRC1), in H4 glioma cells [25] and U87 glioma cells [26], and regulates cancer cell proliferation and apoptosis. SOX2-OT can also act as a destabilizer of transcription factors to control gene expression (Figure 2(c)). A study suggested that SOX2-OT directly binds to the transcription factor FUS and that FUS protein stability is altered by this binding [27]. Thus, SOX2-OT acts as a tumor promoter in pancreatic ductal adenocarcinoma by physically binding to FUS to regulate its downstream cell cycle-associated factors CCND1 and p27 [27] (Figure 1).

## 4. SOX2-OT Is Regulated at the Transcriptional and Posttranscriptional Levels

Most relevant studies have shown that SOX2-OT levels are increased in various cancers and have described the SOX2-OT gene as an oncogene [10]. An increasing number of studies have investigated the mechanism underlying SOX2-OT upregulation in cancer cells (Table 3). These studies have focused on transcriptional and posttranscriptional regulation. Four transcription factors (SOX2, IRF4, AR, and SOX3) were identified to be able to bind directly to the SOX2-OT promoter and promote its transcription (Table 3). Other studies identified three transcriptional inhibitors (NSPc1, MTA3, and YY1) that recruit the repressive complex to the SOX2-OT promoter to repress its expression (Table 3). Interestingly, Shafiee et al. revealed that two miRNAs (miR-211 and miR-375) are responsible for SOX2-OT downregulation in a model of *Helicobacter pylori*-induced carcinogenesis (Table 3, Figure 1).

## 5. SOX2-OT Is Upregulated during Central Nervous System Development, and Its Expression Is Restricted to the Brain

Studies have reported that a striking 40% of lncRNAs are expressed specifically in the brain, indicating the importance of lncRNAs in central nervous system development [28]. Numerous lncRNAs have been identified as regulators of the central nervous system development. Early studies showed that SOX2-OT is highly expressed in mouse embryonic stem cells and is downregulated during the differentiation of embryoid bodies into mesoderm [2]. However, Messemaker et al. demonstrated strong upregulation of SOX2-OT upon the differentiation of embryoid bodies into neuroectoderm, and upregulation of SOX2-OT was found to coincide with neural progenitor/stem cell formation as assessed via the induction of the SOX1 expression, which is a very early and specific marker of the neuroectodermal lineage [7]. Furthermore, SOX2-OT expressed sequence tags have been found in differentiated mouse neural stem cells, and its expression is confirmed in mouse primary neuronal cells [2]. RNA whole-mount in situ hybridization showed that in mice, SOX2-OT expression is limited to the developing brain, the ventral part of the neural tube, and the optic vesicle in mice [7]. Another study indicated that SOX2-OT is expressed in the developing cerebral cortex of mice, where it represses neural progenitor cell proliferation and promotes neuronal differentiation [20].

To investigate the possible involvement of SOX2-OT in neural differentiation processes, Amaral et al. examined the dynamic change in the SOX2-OT expression via a neurosphere assay, an in vitro model of neurogenesis with cultures of neurospheres originating from neural stem cells and undifferentiated precursors in the subventricular zone of adult mice [2]. The differentiated population of neurons and glial cells from neurospheres cultured for 7 days in differentiation medium exhibited increased expression of SOX2-OT [2].

Similar results have also been found in developing zebrafish embryos [7]. Studies have revealed that SOX2-OT is expressed in neuroectodermal tissue in zebrafish embryos at the tailbud stage [2]. Subsequently, SOX2-OT is highly expressed throughout the developing brain and eyes and is expressed at lower levels in the posterior neural tube at 28 hours postfertilization (hpf). In situ hybridization indicated specific expression of SOX2-OT in the retina and central nervous system in 48 hpf embryos, and this expression was maintained in the brain throughout the embryonic development until at least 6 days postfertilization (dpf) [2].

Importantly, data from the Genotype-Tissue Expression (GTEx) project show that in adult humans, the SOX2-OT expression is almost completely restricted to the brain, including regions such as the cortex, hippocampus, hypothalamus, cerebellum, and spinal cord [29]. Single-cell RNA-seq data in the Human Cell Landscape (HCL) project indicate that SOX2-OT expression is concentrated in oligodendrocytes and FGF13^+^ or CXCL14^+^ neurons in adult humans [30].

In summary, SOX2-OT is upregulated during central nervous system development (neurogenesis), and its expression is ultimately restricted to the brain in adult vertebrates.

## 6. SOX2-OT SNPs Are Associated with Mental Illnesses

Because SOX2-OT expression is restricted to the brain in adult humans, SOX2-OT SNPs are correlated with various mental illnesses, as identified by various studies. Genome-wide association studies (GWAS) indicate that the SNPs mapped to the SOX2-OT gene are associated with mental illnesses such as schizophrenia, general cognitive disorders, insomnia, eating disorders, night sleep phenotypes, and anorexia nervosa (Table 4). More than 50% of SOX2-OT-associated diseases are mental illnesses (Table 4). Interestingly, almost all SOX2-OT SNPs are located in the intronic region of the SOX2-OT gene, possibly because the SOX2-OT gene encompasses a genomic region of more than 750 kb. However, one mutation (rs75380963) is located in the exonic region of the SOX2-OT gene (Table 4). Some of the mutations, for example, rs2567646 (general cognitive disorders), rs2216428 (general cognitive disorders), rs4854912 (eating disorders in patients with bipolar disorder), and rs13086738 (eating disorders in patients with bipolar disorder), are strongly correlated with mental illnesses, with odds ratios (ORs) of greater than 1.5 (Table 4, Figure 1).

In contrast to the evidence supporting the relationship between SOX2-OT and mental illnesses, evidence for the association between SOX2-OT SNPs and cancers is scarce. We found no data regarding the association between SOX2-OT SNPs and cancers in the Catalogue of Somatic Mutations in Cancer (COSMIC) or The Cancer Genome Atlas (TCGA) Program database. However, one study demonstrated that a SOX2-OT SNP (rs9839776) is strongly associated with increased expression of SOX2-OT in breast cancer tissues and that this SNP increases the risk of breast cancer in Chinese women (OR: 1.42; 95% CI: 1.06-1.90; *p* = 0.018) [31]. In addition, another study revealed that copy number alteration (CNA) in the SOX2-OT locus is associated with esophageal squamous cell carcinoma [32].

## 7. SOX2-OT Is an Oncogene and a Biomarker for Cancers

lncRNAs have been demonstrated to be upregulated or downregulated during tumorigenesis and to function as oncogenes, suppressors, clinically useful diagnostic/prognostic biomarkers, or therapeutic targets in cancers because of their high sensitivity and specificity [33]. Accumulating evidence indicates that SOX2-OT is a key regulator of cancer stem cells and participates in cancer progression [10]. SOX2-OT is notably upregulated in numerous tumor tissues and cells (Table 5) and plays a vital role as an oncogene to promote the proliferation, invasion, migration, and growth of cancer cells and to suppress their apoptosis [10]. Depletion of SOX2-OT inhibits tumor cell proliferation, migration, invasion, and EMT [10]. However, a study showed that SOX2-OT is downregulated in gastric cancer, which contradicts the findings of the other four studies (Table 4). This contradictory result may have occurred because SOX2-OT has multiple splice variants. Indeed, Wang et al. thoroughly summarized recent studies regarding SOX2-OT expression, function, regulatory mechanisms, and clinical utility in human cancers [10].

SOX2-OT has been identified as a novel lncRNA that can serve as a prognostic biomarker for cancers. A high level of SOX2-OT correlates well with poor clinical outcomes in cancers [34–45]. Li et al. performed a meta-analysis of 13 selected studies by a comprehensive search of PubMed, EMBASE, Cochrane Library, and TCGA and found that the elevated SOX2-OT expression is significantly related to shorter overall and disease-free survival times in cancer patients [45]. Cancer patients with high SOX2-OT expression are more likely to have an advanced clinical stage, earlier lymphatic metastasis, earlier distant metastasis, a larger tumor size, and more extreme tumor invasion than those with low SOX2-OT expression [45]. In addition, two other meta-analyses consistently demonstrated that high SOX2-OT expression is significantly associated with worse overall survival, advanced clinical stage, worse tumor differentiation, earlier distant metastasis, and earlier lymph node metastasis in various cancers [39, 41, 46]. SOX2-OT expression could thus be a promising prognostic biomarker for poor survival in a variety of cancers.

In addition to its prognostic value, circulating or exosome-derived SOX2-OT exhibits diagnostic value in non-small-cell lung cancer and lung squamous cell carcinoma [43, 44, 47]. Kamel et al. demonstrated that circulating SOX2-OT can distinguish non-small-cell lung cancer patients from control individuals, with an area under the curve of 0.73 (76.3% sensitivity and 78.6% specificity) [44]. Moreover, the combination of GAS5 expression and SOX2-OT expression can differentiate non-small-cell lung cancer patients from control individuals with increased sensitivity (83.8) and specificity (81.4) compared with those of SOX2-OT expression alone [44]. Teng et al. analyzed the level of exosomal SOX2-OT in plasma and concluded that the level of exosomal SOX2-OT is significantly increased in lung squamous cell carcinoma patients compared to normal control individuals, indicating the strong power of exosomal SOX2-OT for detecting lung squamous cell carcinoma. In that analysis, the area under the curve was 0.815, and the sensitivity and specificity were 76% and 73.17%, respectively [47]. Thus, SOX2-OT may serve as a promising noninvasive plasma-based diagnostic biomarker for cancers (Figure 1).

## 8. SOX2-OT Mediates Diabetic Complications

A few studies have investigated the possible association of SOX2-OT with diabetic complications, including diabetic nephropathy [12, 13] and diabetic retinopathy [11]. Microarray and bioinformatics analyses indicated that SOX2-OT is significantly downregulated in mice with diabetic nephropathy compared to control mice, and this result was confirmed in cultured human podocytes and mesangial cells [12]. SOX2-OT overexpression significantly alleviates high glucose-induced injury to human podocytes via autophagy induction through the miR-9/SIRT1 axis [13]. Conversely, although the SOX2-OT expression is significantly downregulated in the retinas of mice with streptozocin-induced diabetes, SOX2-OT knockdown protects retinal ganglion cells against high glucose-induced injury in vitro [11].

## 9. SOX2-OT and Other Diseases

In addition to the evidence supporting its involvement in cancers, mental illnesses, and diabetic complications, emerging evidence indicates the association of SOX2-OT with other diseases and events, such as miscarriage [48], septic cardiomyopathy [16], spinal cord injury [49], multiple sclerosis [50], and myopia [51]. An SNP (rs9839776 C>T) in the intronic region of the SOX2-OT gene is associated with increased risk for recurrent miscarriage (CT vs. CC: adjusted OR = 1.357, 95%CI = 1.065 − 1.728, *p* = 0.0134) [48]. In addition, Chen et al. found that SOX2-OT was overexpressed and mitochondrial dysfunction occurred in a mouse model of lipopolysaccharide-induced septic cardiomyopathy; moreover, cardiac-specific knockdown of SOX2-OT via adeno-associated virus 9 (AAV9) harboring SOX2-OT siRNA ameliorated mitochondrial dysfunction in septic cardiomyopathy [16]. A lncRNA PCR array containing 90 common lncRNAs in peripheral blood mononuclear cells from patients with multiple sclerosis revealed a group of dysregulated lncRNAs in multiple sclerosis patients, and SOX2-OT was one of the most strongly downregulated lncRNAs with *p* < 0.001 [50]. However, the SOX2-OT level is not associated with clinical variables such as the disease duration and expanded disability status scale score [50].

## 10. Conclusions and Future Directions

SOX2-OT is upregulated in many cancers and plays an oncogenic role in most tumors. In addition, SOX2-OT is upregulated during central nervous system development and is ultimately restricted to the brain in adult vertebrates. Emerging evidence indicates that multiple factors, including transcriptional activators (SOX2, IRF4, AR, and SOX3) and transcriptional inhibitors (NSPc1, MTA3, and YY1), as well as miRNAs (miR-211 and miR-375), can control the SOX2-OT expression transcriptionally or posttranscriptionally. However, rigorous investigations of the cause and effect mechanism underlying its upregulation in cancers and the central nervous system remain limited.

The downstream targets of SOX2-OT have been elucidated. SOX2-OT performs various molecular and cellular functions via regulation of SOX2 (direct or indirect interactions), regulation of miRNAs (acting as a miRNA sponge), or regulation of transcriptional process (acting as a bridge between epigenetic factors and DNA). However, the precise role of the SOX2-OT gene in neurogenesis, cancers, mental illnesses, and diabetic complications must be systematically investigated and confirmed in a knockout animal model. Currently, no SOX2-OT knockout model is available to demonstrate the essential role of the SOX2-OT gene in neurogenesis and various diseases, because genetic depletion of a lncRNA—especially a lncRNA with multiple exons and transcription start sites, such as SOX2-OT—is difficult. Fortunately, strategies have been applied to generate lncRNA knockout mice, i.e., transcription start site disruption through the insertion of a transcription termination signal and deletion of important gene segments/exons via CRISPR/Cas9 genome editing [52, 53].

Due to the complexity of transcriptional characteristics, including multiple transcription start sites and numerous transcripts in humans and other vertebrates, each transcript may play a unique role in different tissues, embryonic developmental stages, and disease conditions. There is an urgent demand to develop a method to systemically study each transcript under specific conditions. The most recently developed pooled CRISPR screening platform may constitute a good approach for studying the function of each SOX2-OT transcript [54, 55].

SOX2-OT SNPs are associated with mental illnesses, but the precise functions of these SNPs are still obscure. We may need to investigate whether these SNPs alter SOX2-OT expression. In addition, the upregulation of SOX2-OT is correlated with poor outcomes in cancer patients, suggesting its potential function as a diagnostic and prognostic marker in tumors. However, the expression and chemical stability of SOX2-OT in body fluids remain unclear.

The SOX2-OT gene has been widely studied in the past five years, and many important accomplishments have been achieved. However, studies on the SOX2-OT gene are still rare; less than one hundred papers on the SOX2-OT gene have been to date, despite an increasing trend. We still face many challenges, and many aspects of the SOX2-OT gene need to be investigated to provide a foundation for understanding its functions.

## Figures and Tables

**Table 1:** SOX2-OT regulates SOX2 expression.

Regulation	Intermediator	Cell model	Cellular function	Reference
Increase	miR-200c	Bladder cancer	Metastasis and stemness	Zhan et al. [17]
Increase	Unknown	Esophageal squamous cell carcinoma	Metastasis and stemness	Du et al. [56]
Decrease	Unknown	Septic cardiomyopathy	Mitochondrial dysfunction	Chen et al. [16]
No effect	Unknown	Esophageal squamous cell carcinoma	Cell proliferation	Wu et al. [15]
Increase	Unknown	Cholangiocarcinoma	Proliferation and metastasis	Wei et al. [57]
Decrease	Promoter-enhancer loop	Embryonic development	Neural differentiation	Messemaker et al. [7]
Increase	miR-200 family	Pancreatic ductal adenocarcinoma	EMT, stemness, invasion, and metastasis	Li et al. [18]
Decrease	YY1	Embryonic development	Represses neural progenitor proliferation and promotes neuronal differentiation	Knauss et al. [20]
Increase	Unknown	Pancreatic ductal adenocarcinoma	Proliferation and tumor growth	Zhang et al. [19]
Increase	Unknown	Lung cancer	Proliferation, migration, invasion, and stemness	Wang et al. [38]
Increase	Unknown	Breast cancer	Proliferation	Askarian-Amiri et al. [58]

Note: EMT: epithelial-mesenchymal transition; YY1: Yin Yang-1.

**Table 2:** SOX2-OT is a miRNA sponge.

miRNA	Cancer	Target	Cellular function	Reference
hsa-mir-192-5p, hsa-mir-215-5p, hsa-mir-204-5p, hsa-mir-205-5p, hsa-mir-338-3p, hsa-mir-375	Esophageal squamous cell carcinoma	Unknown	Unknown	Tian et al. [23]
miR-200c	Bladder cancer	SOX2	Increases bladder cancer cell stemness and metastasis	Zhan et al. [17]
miR-146b-5p	Nasopharyngeal carcinoma	HNRNPA2B1	Increases proliferation and metastasis; decreases apoptosis	Zhang and Li [59]
miR-369-3p	Prostate cancer	CFL2	Increases proliferation and migration	Wo et al. [60]
miR-363	Ewing's sarcoma	FOXP4	Increases proliferation and invasion; decreases apoptosis	Ma et al. [61]
miR-132	Non-small-cell lung cancer	ZEB2	Increases proliferation, migration, invasion, and EMT	Zhang et al. [62]
miR-211	Pheochromocytoma	MCL-1 isoform 2	Increases cell viability, migration, and invasion; decreases apoptosis and autophagy	Yin et al. [49]
miR-194-5p	Gastric cancer	AKT2	Promotes proliferation, metastasis, invasion, migration, and EMT	Wei et al. [63], Qu et al. [64]
miR-200 family	Pancreatic ductal adenocarcinoma	SOX2	Promotes EMT and stem cell-like properties	Li et al. [18]
miR-194-5p, miR-122	Glioma	SOX3	Increases proliferation, migration, and invasion; decreases apoptosis	Su et al. [65]

Note: EMT: epithelial-mesenchymal transition; Sox2: SRY-box transcription factor 2; HNRNPA2B1: heterogeneous nuclear ribonucleoprotein A2/B1; CFL2: cofilin 2; FOXP4: forkhead box P4; ZEB2: zinc finger E-box binding homeobox 2; MCL1 Isoform 2: myeloid cell leukemia sequence 1 isoform 2; AKT2: AKT serine/threonine kinase 2; SOX3: SRY-box transcription factor 3.

**Table 3:** SOX2-OT is regulated at the transcriptional and posttranscriptional levels.

Factor	Regulatory effect	Cell model	Reference
NSPc1	Represses transcription	Glioma cells	Liang et al. [26]
MTA3	Represses transcription	Esophageal squamous cell carcinoma cells	Du et al. [56]
SOX2	Promotes transcription	Esophageal squamous cell carcinoma cells	Wu et al. [15]
IRF4	Promotes transcription	Cholangiocarcinoma cells	Wei et al. [57]
YY1	Represses transcription	Pancreatic ductal adenocarcinoma cells	Zhang et al. [19]
AR	Promotes transcription	Embryonic neural stem cells	Tosetti et al. [66]
SOX3	Promotes transcription	Glioblastoma stem cells	Su et al. [65]
miR-211	Downregulates expression by directly binding to SOX2-OT	Embryonal carcinoma stem cells (NT-2)	Shafiee et al. [67]
miR-375	Downregulates expression by directly binding to SOX2-OT	Embryonal carcinoma stem cells (NT-2)	Shafiee et al. [68]

Note: NSPc1: nervous system polycomb 1; MTA3: metastasis-associated protein 3; IRF4: interferon regulatory factor 4; YY1L Yin Yang-1; ARL androgen receptor; Sox3: SRY-box transcription factor 3.

**Table 4:** The SNPs of SOX2-OT are associated with various diseases.

SNP	Mapped gene	Context	Disease/abnormality	PubMed ID
rs13096176	SOX2-OT	intron_variant	Schizophrenia	31740837 [69]
rs4855019	SOX2-OT	intron_variant	Schizophrenia	31740837 [69]
rs9841616	SOX2-OT	intron_variant	Schizophrenia	31740837 [69]
rs35788479	SOX2-OT	intron_variant	General risk tolerance	30643258 [70]
rs114600294	SOX2-OT	intron_variant	General risk tolerance	30643258 [70]
rs833268	SOX2-OT	intron_variant	Male-pattern baldness	30573740 [71]
rs12632136	SOX2-OT	intron_variant	Reaction time	29844566 [72]
rs2216428	SOX2-OT	intron_variant	General cognitive disorder	29844566 [72]
rs1345417	SOX2-OT	intron_variant	Excessive hairiness	29895819 [73]
rs60733335	SOX2-OT	intron_variant	Hair color	30595370 [74]
rs2216427	SOX2-OT	intron_variant	Insomnia	30804565 [75]
rs12485391	SOX2-OT	intron_variant	Smoking status	30595370 [74]
rs2567646	SOX2-OT	intron_variant	General cognitive disorder	29844566 [72]
rs9841616	SOX2-OT	intron_variant	Schizophrenia	25056061 [72]
rs1345417	SOX2-OT	intron_variant	Eyebrow thickness	30248107 [76]
rs9841616	SOX2-OT	intron_variant	Schizophrenia	28991256 [77]
rs9859557	SOX2-OT	intron_variant	Schizophrenia	28991256 [77]
rs833270	SOX2-OT	intron_variant	Balding type 1	30595370 [74]
rs77025239	SOX2-OT	intron_variant	Educational attainment	30595370 [74]
rs1805207	SOX2-OT	intron_variant	Body mass index	30595370 [74]
rs9841616	SOX2-OT	intron_variant	Schizophrenia	26198764 [78]
rs1805203	SOX2-OT	intron_variant	Schizophrenia	26198764 [78]
rs1878874	SOX2-OT	intron_variant	Schizophrenia	26198764 [78]
rs13086738	SOX2-OT	intron_variant	Eating disorder in individuals with bipolar disorder	26433762 [79]
rs4854912	SOX2-OT	intron_variant	Bipolar disorder and eating disorder	26433762 [79]
rs1345417	SOX2-OT	intron_variant	Monobrow	27182965 [80]
rs2718791	SOX2-OT	intron_variant	Smoking initiation	30643251 [81]
rs9859557	SOX2-OT	intron_variant	Schizophrenia	30285260 [82]
rs9859557	SOX2-OT	intron_variant	Schizophrenia	30285260 [82]
rs9841616	SOX2-OT	intron_variant	Schizophrenia	30285260 [82]
rs9841616	SOX2-OT	intron_variant	Schizophrenia	30285260 [82]
rs75380963	SOX2-OT	exon_variant	Corneal astigmatism	30306274 [83]
rs77025239	SOX2-OT	intron_variant	Educational attainment	30038396 [84]
rs2718791	SOX2-OT	intron_variant	Educational attainment	30038396 [84]
rs77025239	SOX2-OT	intron_variant	Educational attainment	30038396 [84]
rs9841382	SOX2-OT	intron_variant	Self-reported risk-taking behavior	30271922 [85]
rs9841382	SOX2-OT	intron_variant	Self-reported risk-taking behavior	30181555 [86]
rs9841382	SOX2-OT	intron_variant	Self-reported risk-taking behavior	30181555 [86]
rs4133078	SOX2-OT	intron_variant	Height	30595370 [74]
rs7631379	SOX2-OT	intron_variant	Smoking initiation	30643251 [81]
rs34308817	SOX2-OT	intron_variant	Ankle injury	28957384 [87]
rs6443750	SOX2-OT	intron_variant	Body mass index	30595370 [74]
rs6443750	SOX2-OT	intron_variant	Body mass index	30239722 [88]
rs186834402	SOX2-OT	intron_variant	Interferon gamma levels	27989323 [89]
rs10937060	SOX2-OT	intron_variant	Night sleep phenotypes	27126917 [90]
rs9839776	SOX2-OT	intron_variant	Anorexia nervosa	24514567 [8]
rs4510419	SOX2-OT	intron_variant	Smoking initiation	30643251 [81]
rs9839776	SOX2-OT	intron_variant	Breast cancer	28240100 [31]
rs9839776	SOX2-OT	intron_variant	Recurrent miscarriage	31827385 [48]

Note: SNPs: single-nucleotide polymorphisms; OR: odds ratio.

**Table 5:** Expression status of SOX2-OT in various cancers.

Expression status	Cancer	Reference
Increased	Lung cancer	Hou et al. [34], Zhang et al. [62], Jazi et al. [91]
Decreased	Gastric cancer	Farhangian et al. [92]
Increased	Gastric cancer	Zou et al. [37], Zhang et al. [36], Wei et al. [63], Qu et al. [64]
Increased	Esophageal cancer	Aliereza et al. [93], Tian et al. [23], Wu et al. [15]
Increased	Breast cancer	Iranpour et al. [94], Tang et al. [31]
Increased	Hepatocellular carcinoma	Sun et al. [42], Shi et al. [35]
Increased	Ovarian cancer	Han et al. [95]
Increased	Pancreatic ductal adenocarcinoma	Li et al. [18], Zhang et al. [19]
Increased	Cholangiocarcinoma	Li et al. [40], Wei et al. [57]
Increased	Osteosarcoma	Wang et al. [38]
Increased	Laryngeal squamous cell carcinoma	Tai et al. [24], Feng et al. [96]
Increased	Nasopharyngeal carcinoma	Zhang et al. [59]
Increased	Glioblastoma	Wang et al. [25]
Increased	Bladder cancer	Zhan et al. [17]
Increased	Prostate cancer	Wo et al. [60]
Increased	Ewing's sarcoma	Ma et al. [61]
Increased	Colorectal cancer	Liu et al. [97]
